# Eltrombopag restores T-cell homeostasis in aplastic anemia by regulating oxidative metabolism and reactive oxygen species levels

**DOI:** 10.1007/s00277-026-07060-7

**Published:** 2026-05-18

**Authors:** Ting Wang, Qiulin Chen, Yan Ma, Boyi Wang, Nianbin Li, Yutian Zhang, Rong Fu

**Affiliations:** 1 Department of Hematology, Tianjin Medical University General Hospital, No.154 Anshan Road, Heping District, Tianjin, 300052 China; 2 Tianjin Key Laboratory of Bone Marrow Failure and Malignant Hemopoietic Clone Control, Tianjin, 300052 China; 3 Tianjin Institute of Hematology, Tianjin, 300052 China

**Keywords:** Aplastic anemia, Eltrombopag, T cell, Oxidative metabolism, Immunomodulation

## Abstract

Aplasticanemia (AA) is an autoimmune-mediated bone marrow failure syndrome characterized by excessive T-cell activation and destruction of hematopoietic stem and progenitor cells. Eltrombopag (ELT), a thrombopoietin receptor agonist, has been shown to promote hematopoiesis and exert immunomodulatory effects. However, its role in regulating T cells in AA remains unclear. This study aimed to investigate whether ELT modulates the T-cell functional phenotype via the oxidative metabolism pathway. Using single-cell RNA sequencing of AA mouse models under different treatment regimens, we identified differentially expressed genes in T-cell subsets associated with oxidative stress. ELT treatment effectively reduced reactive oxygen species (ROS) levels in both CD4⁺ and CD8⁺ T cells, reshaped T-cell subset composition, and attenuated cytotoxic activity. Mechanistically, Western blot analysis confirmed that ELT significantly upregulated ENPP1 and ENTPD5, which are involved in oxidative metabolism. Collectively, these findings reveal a previously unrecognized mechanism through which ELT restores T-cell homeostasis by fine-tuning oxidative metabolism, highlighting its therapeutic potential beyond hematopoietic stimulation in AA.

**Supplementary Information:**

The online version contains supplementary material available at 10.1007/s00277-026-07060-7.

## Introduction

Aplastic anemia (AA) is a rare and life-threatening bone marrow failure syndrome, clinically characterized by pancytopenia and bone marrow hypoplasia. Extensive studies have demonstrated that the pathogenesis of AA primarily arises from immune-mediated destruction of hematopoietic stem and progenitor cells (HSPCs), with autoreactive T cells playing a central role in disease progression [1]. Patients with AA typically exhibit disturbances in the T-cell compartment, including an imbalanced CD4⁺/CD8⁺ T-cell ratio, a skewed Th1/Th2 profile, increased Th17 cells, and reduced regulatory T cells (Tregs), reflecting an enhanced inflammatory state and a loss of immune tolerance [2]. Both clinical and experimental studies provide strong evidence supporting the autoimmune pathogenesis of AA. Approximately 70–80% of patients respond to immunosuppressive therapy (IST) [3], bone marrow–derived T cells from AA patients can suppress hematopoietic function in healthy donors in vitro [4]. These findings highlight the pivotal role of T cells in the onset and progression of AA and suggest that restoring immune homeostasis may represent an effective therapeutic strategy.

Eltrombopag (ELT) is an oral, non-peptide thrombopoietin receptor agonist (TPO-RA) that has achieved groundbreaking progress in the treatment of AA in recent years, particularly for patients who are unresponsive to IST or ineligible for bone marrow transplantation [5]. ELT was initially developed to stimulate megakaryopoiesis and platelet production, but subsequent studies have revealed its significant effects in enhancing erythroid and myeloid hematopoiesis and promoting HSPC expansion [6]. Moreover, growing evidence indicates that ELT exerts immunomodulatory effects, including reducing the expression of inflammatory cytokines, regulating immune cell proliferation, and influencing the bone marrow microenvironment [7, 8]. However, the precise mechanisms by which ELT regulates T-cell functional phenotype in AA remain unclear.

Reactive oxygen species (ROS) are key regulators of T-cell activation, differentiation, and effector functions [9]. Under physiological conditions, ROS act as secondary messengers in T-cell receptor (TCR) signaling to modulate immune responses; however, excessive ROS can induce oxidative stress, mitochondrial dysfunction, and immune dysregulation [10]. Recent studies have found that ROS levels are significantly elevated in T cells from AA patients, closely correlating with increased cytotoxicity and enhanced expression of pro-inflammatory cytokines [11, 12]. Therefore, modulating redox homeostasis may represent a potential strategy to restore immune function in AA patients. oxidative metabolism is a key pathway regulating T-cell metabolism and functional phenotype; enhanced oxidative metabolism activity meets the energy and biosynthetic demands of memory T cells and Tregs, whereas effector T cells rely more heavily on glycolytic metabolism [13]. Although oxidative metabolism is closely linked to T-cell functional phenotype, whether ELT modulates ROS levels and T-cell activity through the oxidative metabolism pathway remains largely unexplored.

Therefore, this study integrated single-cell transcriptome sequencing with multi-level functional validation to investigate the regulatory effects and underlying mechanisms of ELT on T cell function in AA.

## Methods

### Establishment of the AA mouse model and drug intervention

Twelve B6D2F1 mice (Beijing Vital River Laboratory Animal Technology Co., Ltd.) were irradiated (4.5 Gy) and infused with C57-derived lymphocytes to induce bone marrow failure [14]. Mice were randomly assigned to four groups: PBS, ELT (40 mg/kg per day), CsA (50 mg/kg per day), and Eltrombopag combined with CsA (ELT + CsA, EC, 40 mg/kg per day + 50 mg/kg per day). Drugs were administered intraperitoneally for 12 consecutive days. Femoral bone marrow cells were harvested at the end of treatment and used for single-cell RNA sequencing.

### Single-cell RNA sequencing and bioinformatics analysis

Bone marrow cells were processed using the 10x Genomics Chromium platform according to the manufacturer’s instructions. Sequencing data were analyzed using Seurat (version 4.0) for quality control, clustering, and differential gene expression analysis. Gene Ontology (GO) and Kyoto Encyclopedia of Genes and Genomes (KEGG) pathway enrichment analyses were subsequently performed.

### Patient and sample collection

Ten newly diagnosed AA patients (5 males, 5 females; median age 48, range 14–72) and four age- and sex-matched healthy controls (2 males, 2 females; median age 42, range 26–68) were recruited at Tianjin Medical University General Hospital between June 2023 and January 2024 [14]. The study was approved by the institutional ethics committee, and written informed consent was obtained from all participants in accordance with the Declaration of Helsinki.

### T-cell isolation, culture, and in vitro eltrombopag treatment

1. Isolation of bone marrow mononuclear cells and CD3⁺ T cells.
  Bone marrow mononuclear cells (BMMCs) were isolated from fresh EDTA-anticoagulated bone marrow by density-gradient centrifugation. CD3⁺ T cells from AA patients and healthy controls were purified by immunomagnetic separation using anti-human CD3 microbeads (Miltenyi Biotec) according to the manufacturer’s instructions. Preparations with a CD3⁺ purity of ≥ 90% were used for subsequent experiments.
2. Expansion of CD3⁺ T cells.
  Purified CD3⁺ T cells were cultured at a density of 0.5–1 × 10⁶ cells/mL in RPMI-1640 medium supplemented with 5–10% fetal bovine serum and penicillin–streptomycin. Cells were activated on Day 0 with anti-CD3 (1 µg/mL), anti-CD28 (1 µg/mL), and recombinant human IL-2 (300 IU/mL), and maintained at 37 °C in a humidified incubator with 5% CO₂. For eltrombopag treatment, ELT (1000 nM) was added on Day 3, and cells were harvested after an additional 48 h for downstream analyses.

### Flow cytometry

Cells were stained for surface markers (CD3, CD8) and intracellular markers (IFN-γ, perforin, IL-4, IL-17, FOXP3) using standard fixation and permeabilization protocols. Intracellular ROS was measured with the DCFDA probe (MedChemExpress). Data were acquired on a Beckman Coulter CytoFLEX and analyzed with CytExpert software. Because CD4 is susceptible to activation-induced internalization, CD4⁺ T-cell subsets were identified by gating on CD3⁺CD8⁻ events rather than CD4⁺ events, thereby avoiding underestimation of activated CD4⁺ T cells.

### Western blotting

Total protein was extracted from T cells using RIPA lysis buffer. FOXO3, ENPP1, ENTPD5, and TRX protein levels were detected by Western blotting, with β-actin used as a loading control. Band intensities were quantified using ImageJ software.

### Statistical analysis

Statistical analyses were performed using SPSS 26.0 and GraphPad Prism 9. Data are presented as mean ± standard deviation (SD). Comparisons between two groups were conducted using Student’s t-test, while multiple-group comparisons were performed using one-way analysis of variance (ANOVA). A P value < 0.05 was considered statistically significant.

## Results

### Preliminary analysis of immune cell subsets in bone marrow

**Fig. 1:**
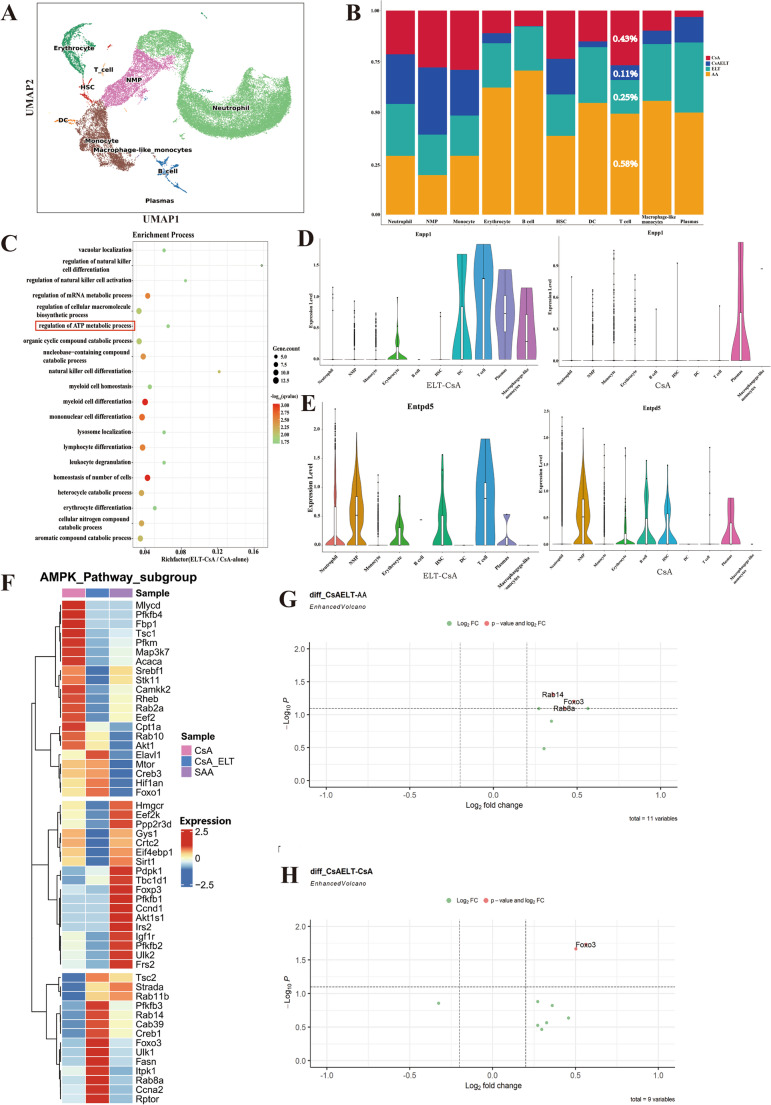
Overview of cellular subsets in untreated and treated AA mouse samples. (**A**)UMAP plot of nine cell clusters, defined by the expression of marker genes across different subsets of single-nucleated cells. (**B**)Bar charts showing the proportions of immune cell subsets in the ELT group, ELT + CsA group, CsA group, and AA group. (**C**)KEGG pathway analysis of differentially expressed T cell genes in the, ELT + CsA group compared with the CsA group. (**D**)Violin plot showing the expression of the highly variable gene ENPP1 in T cells in the ELT + CsA group and the CsA group. (**E**)Violin plot illustrating the expression of the highly variable gene ENTPD5 in T cells in the ELT + CsA group and CsA group. (**F**)Heatmap showing the correlation between AA group, ELT + CsA group, CsA group, and the AMPK gene signature. (**G**)Volcano plot depicting differentially expressed genes in the T cell AMPK pathway in the ELT + CsA group compared to the CsA group (criteria: *p* < 0.05, log2FC > 0.5). (**H**) Volcano plot of differentially expressed genes in the T cell AMPK pathway in the ELT + CsA group compared to the CsA group (criteria: *p* < 0.05, log2FC > 0.5)

In this study, twelve B6D2F1 mice were divided into four groups for AA modeling. Femoral bone marrow cells were collected and subjected to 10x Chromium single-cell RNA sequencing. Data quality control was performed using the Seurat R package, and 30 distinct cell clusters were identified via t-distributed stochastic neighbor embedding (t-SNE) analysis. Based on the expression of marker genes reported in the literature and annotations from the SingleR package (v2.0.0), all cells were classified into nine major cell types (Fig. 1A).

Overall comparison of immune cell subsets across the AA and treatment groups showed that the proportion of T cells was lowest in the ELT + CsA group (0.11%), whereas higher proportions were observed in the AA (0.58%), ELT (0.25%), and CsA (0.43%) groups (Fig. 1B). To further explore the effects of ELT and CsA on T-cell function, differential gene enrichment analysis was conducted between the CsA and ELT + CsA groups. It was found that the differentially expressed genes were enriched in pathways including RNA catabolism, regulation of myeloid homeostasis, metabolism of cellular nucleotide-binding proteins, and oxidative phosphorylation pathways associated with ATP level regulation. We focus on oxidative phosphorylation pathway (KEGG ID: hsa00190)(Fig. 1C).Notably, ENPP1 and ENTPD5 exhibited differential expression in T-cell subsets between the CsA and ELT + CsA groups (Fig. 1D and E).

Further single-cell RNA sequencing analysis using the KEGG database identified AMPK as a key regulatory pathway of oxidative metabolism (Fig. 1F). Comparative analysis among the AA, ELT + CsA, and CsA groups revealed enrichment of 14 genes in the ELT + CsA group, 21 in the AA group, and 18 in the CsA group. Differential analysis of the AMPK pathway showed a significant association between ELT treatment and FOXO3 expression (Fig. 1G and H).

### ELT regulates immune function of CD8⁺ and CD4⁺ T cells in AA via the oxidative metabolism pathway

#### ELT reduces ROS levels in CD8⁺ and CD4⁺ T cells from AA patients

**Fig. 2:**
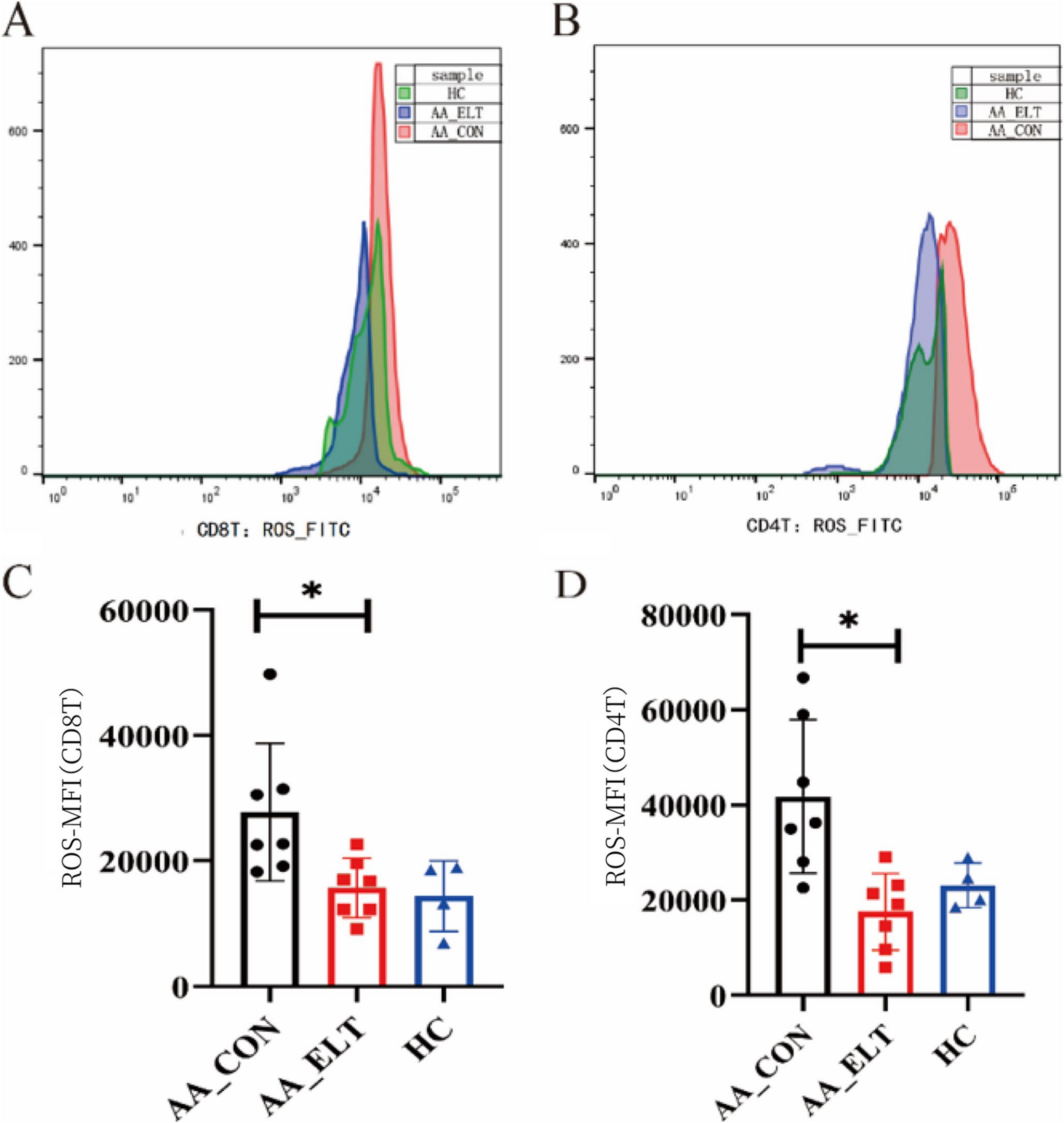
ELT reduces ROS levels in CD8⁺ and CD4⁺ T cells. (**A**) Representative flow cytometry histograms showing ROS levels (FITC intensity) in CD8⁺ T cells from from HC, AA, and AA_ELT groups (**B**) Representative flow cytometry histograms showing ROS levels in CD4⁺ T cells (**C**) Quantitative analysis of ROS mean fluorescence intensity (MFI) in CD8⁺ T cells among groups. (**D**) Quantitative analysis of ROS MFI in CD4⁺ T cells among groups. Data are presented as mean ± SD. (*P < 0.05)

To validate the immunoregulatory effects of ELT on CD8⁺ and CD4⁺ T cells, intracellular ROS levels were measured in T cells from the bone marrow of AA patients and healthy controls (HCs). Compared with HCs, CD8⁺ T cells from AA patients showed elevated ROS levels (27,756.84 ± 10,988.34 vs. 14,391.33 ± 5,597.22), though the difference was not statistically significant (*P* = 0.094). ELT treatment significantly reduced ROS levels in CD8⁺ T cells (27,756.84 ± 10,988.34 vs. 15,651.57 ± 4,698.89, P = 0.0187) (Fig. 2A and C).

Similarly, ROS levels in CD4⁺ T cells were higher in AA patients compared with HCs (41,744.34 ± 16,144.08 vs. 23,054.60 ± 4,694.40, *P* = 0.053), and ELT treatment significantly decreased ROS levels in CD4⁺ T cells (41,744.34 ± 16,144.08 vs. 17,502.79 ± 8,045.54, *P* = 0.0115) (Fig. 2B and D), suggesting that ELT alleviates oxidative stress in T cells from AA patients.

#### ELT modulates the proportion and functional phenotype of CD8⁺ T-cell subsets in AA patients

To further examine the effects of ELT on CD8⁺ T-cell functional phenotype, CD3⁺ T cells from untreated AA patients were expanded and activated in vitro. Experimental groups included untreated AA, ELT-treated, and Temple-treated cells (Temple, a superoxide dismutase mimetic, as a positive control).

**Fig. 3:**
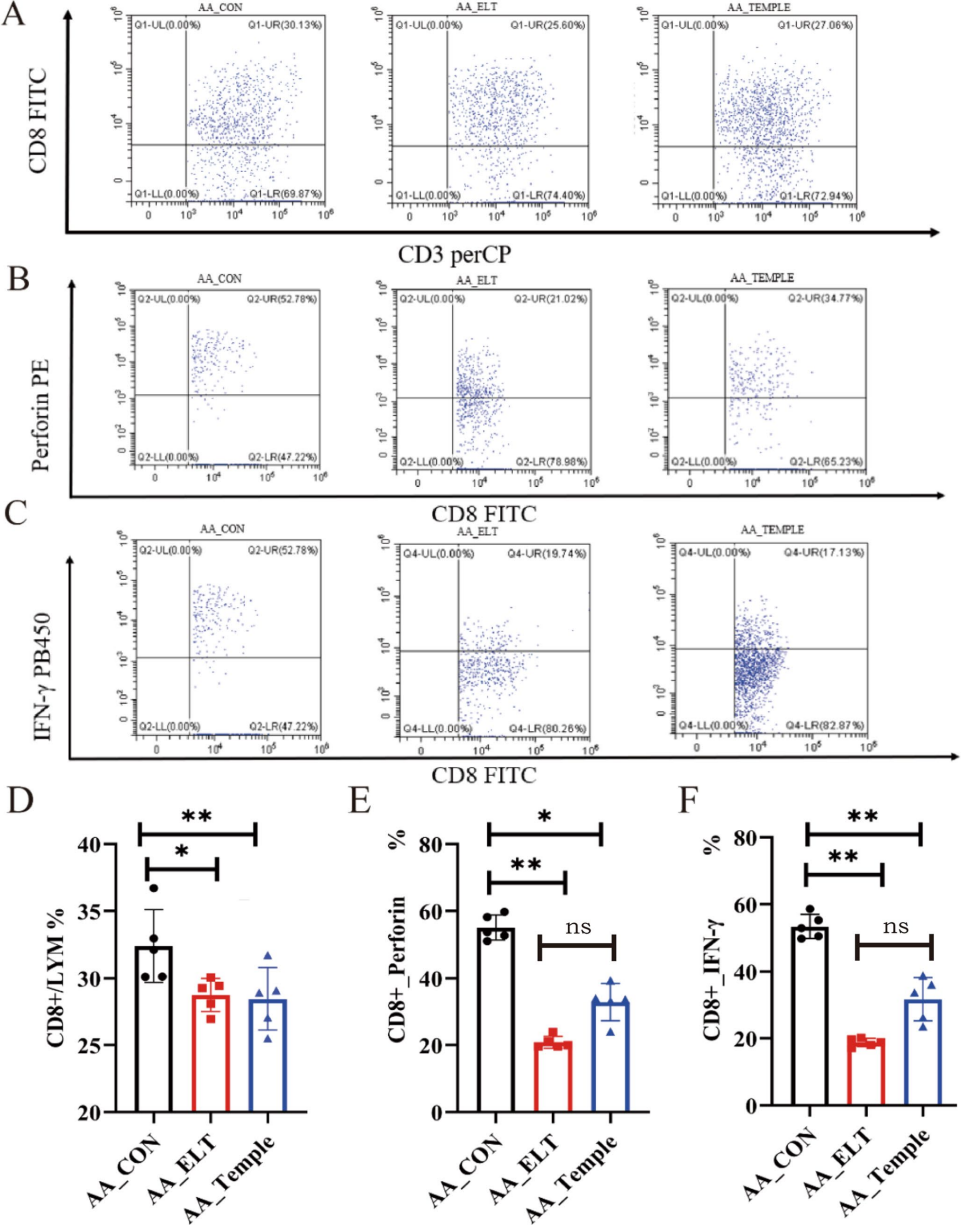
ELT modulates the expansion ratio and functional phenotype of CD8⁺ T cells in vitro. (**A**) Percentage of CD8⁺ T after restimulating with anti-CD3/28 beads in AA, ELT-treated, and Temple-treated groups (**B**) Percentage of Perforin-positive cells within the CD8⁺ T cell population in AA, ELT-treated, and Temple-treated groups (**C**) Percentage of IFN-γ positive cells within the CD8⁺ T cells population in AA, ELT-treated, and Temple-treated groups(**D**)Comparison of CD8⁺ T cells proportion across treatment groups, Perforin + and IFN-γ + CD8⁺ T cells population in across treatment groups. Data are presented as mean ± SD. (*P < 0.05, ** *P* < 0.01)

Compared with the AA group, the proportion of CD8⁺ T cells (CD8⁺T/Lym%) in the ELT group was significantly reduced (32.40 ± 2.70% vs. 28.76 ± 1.23%, *P* = 0.018) (Fig. 3A and D). There was no significant difference between the ELT and Temple groups (28.46 ± 2.34% vs. 28.76 ± 1.23%, *P* = 0.6806), suggesting that ELT may have antioxidant effects similar to Temple.

To assess CD8⁺ T-cell functional phenotype, perforin and IFN-γ expression were measured. The proportion of perforin⁺ CD8⁺ T cells was significantly decreased in the ELT group (53.45 ± 3.58% vs. 18.79 ± 1.22%, *P* = 0.007) (Fig. 3B and E), and IFN-γ⁺ CD8⁺ T cells were also reduced compared with the AA group (55.14 ± 3.69% vs. 20.83 ± 1.82%, *P* = 0.008) (Fig. 3C and F). No significant differences were observed between ELT and Temple groups in perforin⁺ (20.83 ± 1.82% vs. 31.73 ± 6.53%, P = NS) or IFN-γ⁺ (18.79 ± 1.22% vs. 32.87 ± 5.55%, *P* = 0.085) CD8⁺ T-cell proportions, indicating that ELT may reduce CD8⁺ T-cell cytotoxicity by modulating oxidative metabolism and ROS levels similar to the SOD mimetic Temple.

#### ELT modulates the proportion and functional phenotype of CD4⁺ T-cell subsets in AA patients

**Fig. 4:**
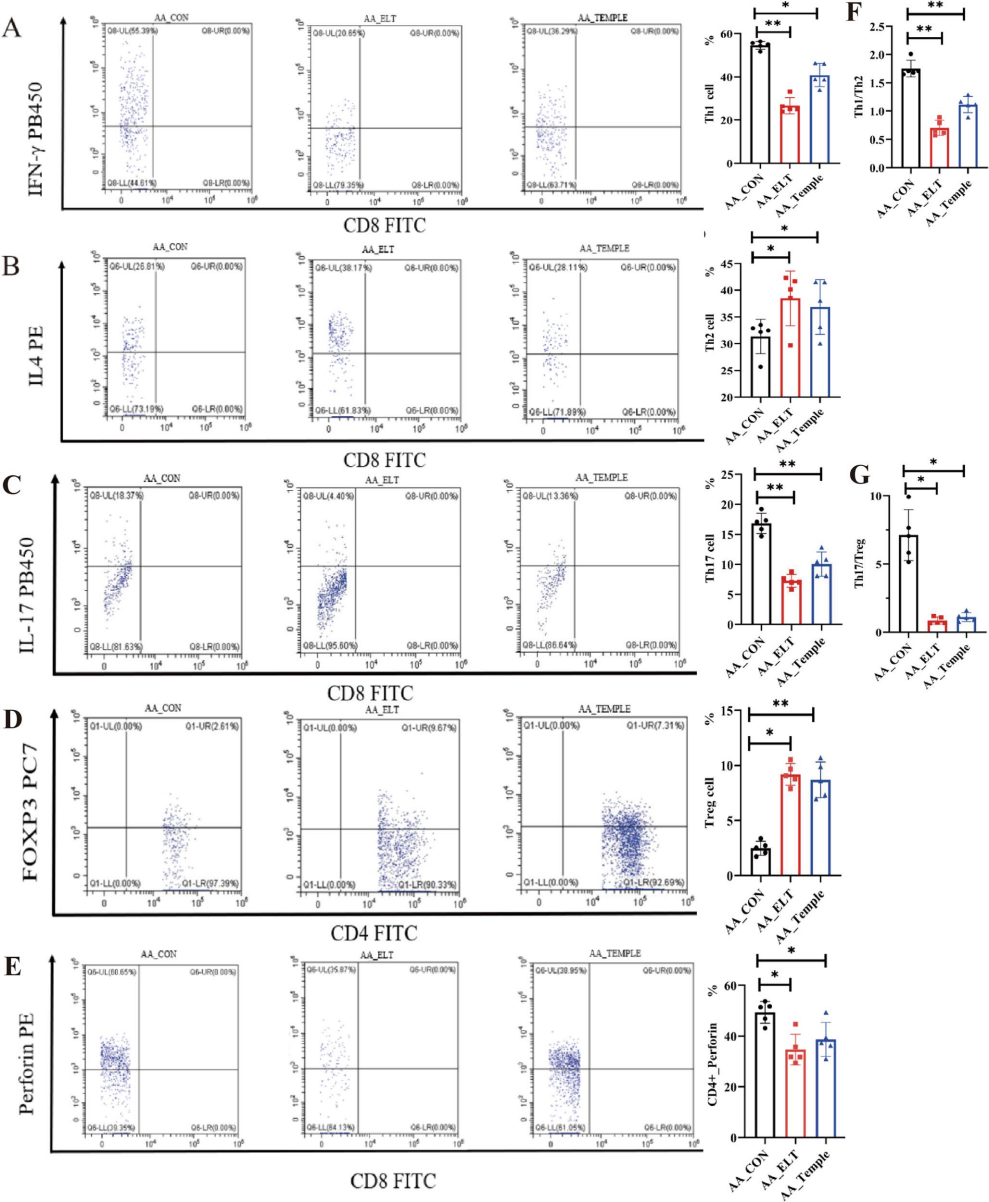
ELT regulates CD4⁺ T-cell subsets, Th1/Th2 and Th17/Treg ratios, and perforin expression in AA, ELT-treated, and Temple-treated groups.(**A**)Percentage of IFN-γ + Th1s among groups. (**B**) Percentage of IL-4⁺ Th2s among groups. (**C**) Percentage of IL-17 A+ Th17s among groups. (**D**) Percentage of FOXP3⁺ Tregs among groups. (**E**) Percentage of Perforin+CD8-T cells among groups. (F)Th1/Th2 ratio among groups (G)Th17/Treg ratio among groups. Data are presented as mean ± SD. ༈*P < 0.05, ** *P* < 0.01༉

To evaluate the effect of ELT on CD4⁺ T-cell functional phenotype, after expanded and activated in vitro of AA, ELT-treated, and Temple-treated groups, the ratios of IFN-γ⁺ Th1 and IL-4⁺ Th2 cells were analyzed. ELT treatment significantly decreased the proportion of IFN-γ⁺ CD4⁺ T cells (54.62 ± 1.81% vs. 26.70 ± 3.80%, *P* = 0.0259) (Fig. 4A), with no significant difference compared to the Temple group (*P* = 0.0632). The proportion of IL-4⁺ CD4⁺ T cells in the ELT group was significantly higher than that in the AA group (38.18 ± 5.10% vs. 31.35 ± 3.19%, *P* = 0.0031) (Fig. 4B), with no significant difference relative to the Temple group. The Th1/Th2 ratio was significantly reduced in the ELT group (0.70 ± 0.13 vs. 1.75 ± 0.15, *P* = 0.0045), suggesting that ELT promotes a Th2-skewed immune response (Fig. 4F).

Further analysis revealed that the proportion of IL-17⁺ CD4⁺ T cells was significantly higher in the AA group than in the ELT group (16.83 ± 1.69% vs. 7.26 ± 1.07%, *P* = 0.0013) (Fig. 4C), whereas no significant difference was observed between ELT and Temple groups. The proportion of FOXP3⁺ Tregs was significantly higher in the ELT group compared with the AA group (9.18 ± 0.99% vs. 2.48 ± 0.63%, *P* = 0.0185) (Fig. 4D), with no significant difference from the Temple group. Consequently, the Th17/Treg ratio in the ELT group was markedly lower than in the AA group (0.87 ± 0.26 vs. 7.12 ± 1.85, *P* = 0.0013) (Fig. 4G), indicating that ELT promotes a balance between inflammatory and regulatory T cells.

Perforin, a key cytotoxic molecule in T-cell–mediated immune responses, was significantly reduced in CD4⁺ T cells after ELT treatment (34.68 ± 6.05% vs. 49.36 ± 4.29%, *P* = 0.0285) (Fig. 4E), with no significant difference compared to the Temple group, suggesting that ELT may attenuate CD4⁺ T-cell cytotoxicity and exert immunomodulatory effects.

### ELT Enhances expression of oxidative metabolism pathway proteins in T cells

Previous experiments confirmed that ELT modulates ROS levels and affects T-cell subset differentiation. Single-cell sequencing data further identified ENPP1, ENTPD5, and FOXO3 as differentially expressed genes between the ELT + CsA and CsA groups. These proteins are key regulators of the oxidative metabolism pathway and can directly or indirectly reduce ROS levels.

**Fig. 5:**
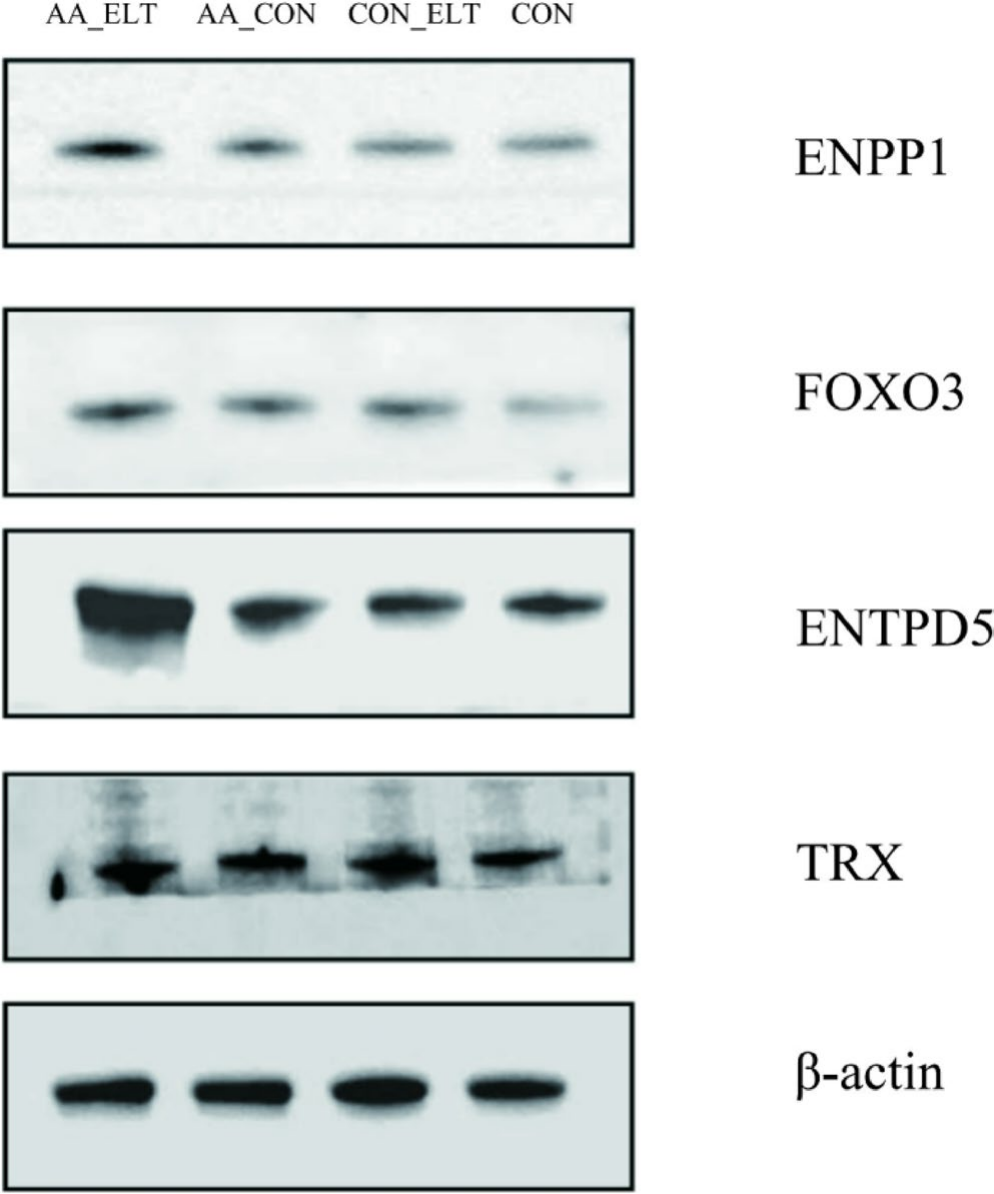
Expression of ENPP1, FOXO3, ENTPD5, and TRX proteins in CD3^+^ T cells from AA patients or healthy controls, with or without ELT treatment by western blot

To validate these findings, CD3⁺ T cells were isolated from the bone marrow of AA patients and healthy controls and cultured in vitro. Upon ELT treatment, Western blot analysis demonstrated significant upregulation of ENTPD5, ENPP1, and the downstream effector TRX, with more pronounced effects observed in T cells derived from AA patients (Fig. 5). However, FOXO3 expression was not significantly altered under our experimental conditions in AA. These results may suggest that ELT plays a crucial role in regulating aberrant oxidative metabolism activity in T cells by specifically upregulates key enzymes such as ENPP1 and ENTPD5, which are involved in redox regulation.

Collectively, these findings demonstrate that ELT modulates T-cell functional phenotype through the oxidative metabolism pathway, which may contribute to its immunoregulatory effects in AA.

## Discussion

This study systematically elucidates a novel mechanism by which ELT exerts immunoregulatory effects in the context of AA through modulation of the oxidative metabolism pathway. By integrating single-cell RNA sequencing, functional assays, and protein expression analyses, we demonstrate that ELT could reduce ROS levels in CD4⁺ and CD8⁺ T cells, restores the balance of T-cell subsets, and attenuates cytotoxic activity. These effects are closely linked to ELT-mediated upregulation of redox-regulatory factors including ENPP1 and ENTPD5, along with their downstream antioxidant effector TRX. These findings not only expand the traditional understanding of ELT as a hematopoietic stimulator but also highlight its significant immunomodulatory potential in AA.

The core pathological mechanism of AA is immune-mediated destruction of HSPCs, in which aberrantly activated T cells play a pivotal role [1]. Numerous studies have shown that AA patients often exhibit an increased proportion of cytotoxic CD8⁺ T cells, skewing toward Th1 and Th17 responses, along with a decreased proportion of Tregs, resulting in loss of immune tolerance and persistent inflammation [2]. Our findings differ from some clinical studies, such as the Phase 2 trial published in NEJM in 2012. In that study, no significant changes were observed in the absolute counts of T cell subsets in patients with refractory aplastic anemia (AA) treated with eltrombopag (ELT). However, our study focused more on the relative balance and functional activity of CD8⁺ and CD4⁺ T cells, rather than their absolute numbers [15]. ELT treatment significantly reduced ROS levels, suggesting its ability to alleviate oxidative stress–driven immune imbalance [16]. It also improves regulatory T-cell functional phenotype [17], and suppresses T-cell activation [18].

T-cell activation and differentiation are closely linked to their metabolic state: effector T cells primarily rely on glycolysis, whereas memory T cells and Tregs depend more on mitochondrial oxidative metabolism for energy [19]. ROS, a key byproduct of oxidative metabolism, functions as an important signaling molecule that regulates immune responses under physiological levels, but excessive ROS leads to oxidative stress and immune dysfunction [20]. Single-cell sequencing in this study revealed that ELT modulates the AMPK–oxidative metabolism signaling pathway in T cells and enriches for genes associated with ATP production and redox homeostasis, notably upregulating ENPP1 and ENTPD5. These molecules are involved in mitochondrial function [21, 22] and antioxidant defense [23], and together with enhanced TRX expression [24], collectively improve T-cell metabolic stability and antioxidative capacity.

ELT not only modulates ROS of T cells in AA patients but also improves their functional phenotype. In CD8⁺ T cells, ELT treatment markedly reduced perforin and IFN-γ expression. In CD4⁺ T cells, ELT decreased the proportions of Th1 and Th17 cells while increasing Th2 and Treg populations, restoring the balance of Th1/Th2 and Th17/Treg ratios. These effects resemble those observed in the ROS scavenger positive control group, suggesting that ELT’s immunoregulatory function may be partly mediated through its ability to modulate redox status.

Our findings indicate that the therapeutic effects of ELT in AA may extend beyond hematopoietic stimulation, directly restoring immune homeostasis by modulating aberrant T-cell functional phenotype. Considering that immune abnormalities may persist in AA patients even after IST [25], the dual actions of ELT—promoting hematopoiesis and regulating immunity—may underlie the higher hematologic response rates observed in combination therapy. Moreover, elevated ROS has been implicated in various autoimmune and inflammatory diseases, highlighting the potential of ELT’s antioxidant and metabolic regulatory properties in other clinical contexts.

This study has several limitations. First, the patient sample size was limited, and heterogeneity in disease severity as well as prior treatment history may have influenced the immunological readouts. Second, although ENPP1 and ENTPD5 were significantly upregulated, FOXO3 expression did not show a statistically significant change. This may be attributed to the fact that FOXO3 activation is not solely dependent on increased total protein levels, as its activity can also be regulated by post-translational modifications such as dephosphorylation and nuclear translocation—mechanisms not fully explored in this study. Furthermore, the precise molecular pathways through which ELT regulates T-cell oxidative metabolism via the TPO receptor signaling remain to be clarified.

## Conclusion

In summary, this study reveals a novel mechanism by which ELT restores T-cell homeostasis in AA through oxidative metabolism modulation, ROS reduction, and T-cell subset rebalancing. These findings deepen our understanding of ELT’s immunoregulatory effects and provide a theoretical basis for dual-targeted therapeutic strategies in AA. Future studies with larger patient cohorts should validate these mechanisms and explore metabolic pathway–targeted interventions to optimize AA treatment.

## Supplementary Material

Below is the link to the electronic supplementary material.

Supplementary Material 1 (DOCX 291 KB)

## Data Availability

If you have any reasonable questions, please contact the corresponding author (furong8369@tmu.edu.cn).
