# Supplementary material for: Eltrombopag restores T-cell homeostasis in aplastic anemia by regulating oxidative metabolism and reactive oxygen species levels
Source: Ann Hematol. 2026 May 18;105(8):323. doi: 10.1007/s00277-026-07060-7 (PMC13346318; doi:10.1007/s00277-026-07060-7)
Supplement: Supplementary file 1 — Supplementary Material 1 (DOCX 291 KB) [file 277_2026_7060_MOESM1_ESM.docx]

Supplementary Material Description

Supplementary Figure 5 Uncropped Western blot images (related to Figure 5). The panels below show full-length blots of FOXO3, ENPP1, ENTPD5, TRX and β-actin, respectively. Prestained molecular weight markers (kDa) are indicated on both sides. All samples were CD3⁺ T cells isolated from AA patients and treated with ELT for 48 h.

AA-ELT AA-CON CON-ELT CON

10kDa

25kDa

25kDa

35kDa

45kDa

60kDa

75kDa

100kDa kDa kDa

100kDa kDa kDa

140kDa

ENTPD5 51kDa

FOXO3 75kDa

ENPP1 131kDa


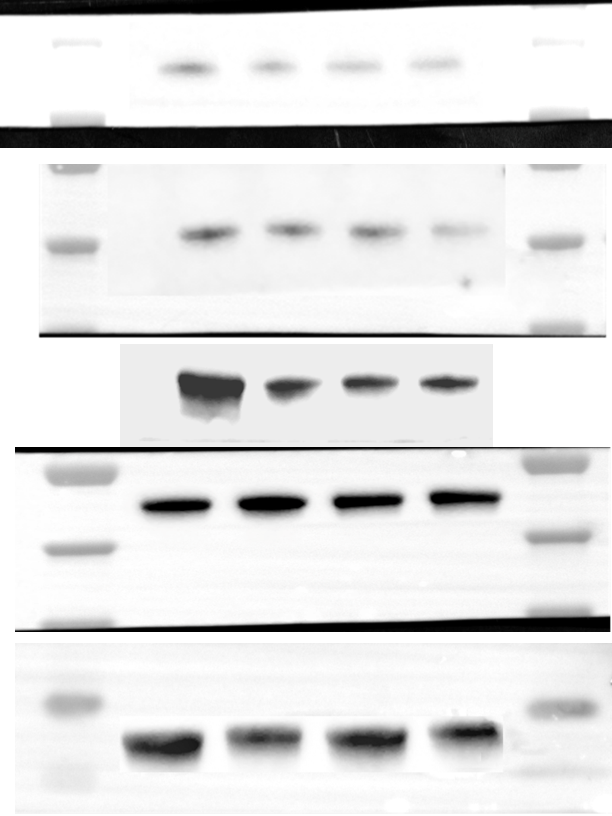


β-actin 37kDa

TRX 12kDa

We have now performed comprehensive densitometric analysis of all Western blot bands using ImageJ software. The detailed results are as follows:

FOXO3 expression analysis: The relatively values were as follows: AA-ELT group 1.41; AA-CON group 1.59; CON+ELT group 2.23; CON group 1.00. The data indicate that ELT treatment did not lead to a significant change in FOXO3 expression within the AA patient group (1.41 vs 1.59, P >0.05).

Complete quantitative data for all OXPHOS-related proteins: To provide full transparency, we have included the complete densitometric analysis for all proteins examined:

| Protein | AA-ELT | AA-CON | CON+ELT | CON |
| --- | --- | --- | --- | --- |
| ENPP1 | 2.43 | 1.45 | 2.04 | 1.00 |
| ENTPD5 | 3.14 | 1.51 | 1.96 | 1.00 |
| TRX | 1.63 | 1.27 | 2.40 | 1.00 |
| FOXO3 | 1.41 | 1.59 | 2.23 | 1.00 |

The results showed that ELT treatment significantly upregulated the expression of ENPP1 and ENTPD5 in T cells derived from AA patients, supporting their roles as key regulators of the OXPHOS pathway. In addition, TRX expression was also increased, indicating enhanced antioxidant capacity and improved redox homeostasis in T cells following ELT treatment. However, FOXO3 expression did not show a significant change, which is consistent with our revised interpretation.
